# Attention-Deficit/Hyperactivity Disorder Symptoms and Anger and Aggression in Russian Adolescents

**DOI:** 10.1016/j.jaacop.2024.01.006

**Published:** 2024-02-16

**Authors:** Johan Isaksson, Denis G. Sukhodolsky, Roman Koposov, Andrew Stickley, Mia Ramklint, Vladislav Ruchkin

**Affiliations:** a Uppsala University, Uppsala, Sweden; b Karolinska Institutet, Stockholm, Sweden; c Yale University School of Medicine, New Haven, Connecticut; d Sala Forensic Psychiatric Clinic, Sala, Sweden; e The Arctic University of Norway, Tromsö, Norway, and Sechenov First Moscow State Medical University, Moscow, Russia; f Södertörn University, Sweden, and National Institute of Mental Health, National Center of Neurology and Psychiatry, Tokyo, Japan

**Keywords:** ADHD, aggression, anger, comorbidity, sex differences

## Abstract

**Objective:**

Aggression is a multifaceted behavior that involves cognitive, behavioral, and affective components. Although aggressive behaviors are commonly observed among individuals with attention-deficit/hyperactivity disorder (ADHD), potential sex-specific aspects of the association between ADHD symptoms and different components of aggression need to be evaluated, while also controlling for comorbid problems.

**Method:**

In the present cross-sectional study, self-reported data were collected from 2,838 adolescents (mean age = 14.89 years) from Russia on ADHD symptoms and cognitive (anger rumination, aggressive beliefs), behavioral (physical, verbal, social, proactive), and affective (trait anger) aspects of aggression as well as comorbid emotional and conduct problems. Generalized linear model analyses were used to examine the associations between ADHD symptoms and aggression and to explore sex differences, while also adjusting for comorbid problems.

**Results:**

Clinically significant levels of ADHD symptoms were associated with all components of aggression, and the associations remained significant after adjusting for emotional and conduct problems. Overall, females had higher levels of trait anger and anger rumination, whereas males had higher levels of aggressive beliefs, proactive aggression, and physical and verbal aggression. There was also a sex-specific association, where males with ADHD symptoms reported higher levels of social aggression. Conduct problems moderated the association between ADHD and aggression, increasing the likelihood of trait anger and social aggression in adolescents without ADHD symptoms.

**Conclusion:**

Clinically significant levels of ADHD symptoms seem to impact all components of aggression independent of comorbidity. It is therefore important to consider aggression when evaluating and treating ADHD.

## Plain language summary

This study investigated the association between self-rated attention-deficit/hyperactivity disorder (ADHD) symptoms and different components of aggression among Russian adolescents. ADHD was related to cognitive, behavioral, and affective aspects of aggression, even when taking emotional and conduct problems into consideration. The findings stress the importance of considering aggression when evaluating and treating ADHD.

Attention-deficit/hyperactivity disorder (ADHD) affects 5% to 7% of children and adolescents^1^ and represents one of the most common neurodevelopmental conditions in youth. Children with ADHD often display functional impairment in such areas as relationships with family and friends and functioning in school,^2^ which decreases their chances of successful social adjustment over time. Although aggression is not pathognomonic for ADHD, it may further negatively impact everyday functioning of children^3^^,^^4^ and their long-term prognosis and represents one of the main reasons for referral for mental health evaluation.^5^

Aggression is commonly defined as any verbal or physical behavior directed toward another person with the intention to cause harm^6^ and involves cognitive, behavioral, and affective components.^7^ The cognitive component of aggression often refers to thoughts, beliefs, and perceptions that influence aggressive behavior; the affective component relates to emotional aspects such as anger; and the behavioral component refers to observable actions that can take various forms, including physical, verbal, or relational aggression. The distinctions between such components are, however, somewhat arbitrary. For instance, while anger is regarded as an emotion that can be defined as a negative feeling state,^8^ anger rumination (eg, excessive thinking about anger experiences) is considered a separate thought process.^9^ A well-known approach draws a distinction between reactive and proactive aggression, as follows^10^: reactive aggression (also sometimes referred to as impulsive or hot aggression) refers to responses to triggers, whereas proactive aggression (instrumental, goal-oriented, or cold-blooded aggression) relates to deliberate/bullying behavior to hurt others or to achieve goals or personal benefit.

Children with ADHD are at risk of demonstrating a range of aggressive behaviors.^4^^,^^5^ More specifically, research suggests that youth with ADHD may differ from their peers in their expression of aggression, primarily in the tendency to respond with anger to provocation, possibly as a result of their greater emotional reactivity.^11^ Similarly, reactive or impulsive aggression may be more closely related to ADHD than proactive aggression,^5^^,^^12^^,^^13^ while social/relational aggression may be disproportionately elevated in the context of ADHD^14^ or be unrelated to ADHD symptoms.^15^ Interestingly, findings regarding the cognitive processes commonly associated with aggression have also been ambiguous in individuals with ADHD. While a wish to retaliate after provocation seems to be more long lasting,^16^ suggesting a tendency to ruminate over anger/frustration experiences, aspects such as hostile attribution bias^17^ and interpretation of facial affect^18^ have not differed from those in controls. Hence, while it is important to know whether the generally observed differences in aggression between individuals with ADHD and controls are applicable to all subtypes of aggression, research findings addressing this issue have been limited and in some respects inconsistent, and there has been no consistent focus on different forms of aggression.

The severity of aggression in children and adolescents with ADHD has been linked both to the severity of ADHD and to its comorbidity.^19^ Of the comorbid conditions, research has primarily indicated conduct disorder (CD) and oppositional defiant disorder, with 20% and 60% comorbidity rates, respectively,^20^ which in combination with ADHD increase the risk of impulsive aggression in response to low levels of provocation and of wanting to retaliate over a longer period of time after provocation.^16^ At the same time, it has been suggested that children with ADHD show high levels of aggression, even after controlling for comorbid conduct problems^5^ and although there is considerable overlap between the conduct problems and ADHD domains, they have also been shown to differ in several important aspects.^21^ There is also some evidence that comorbid anxiety may increase the risk for anger in adults with ADHD.^22^ Despite a substantial degree of comorbidity between ADHD and emotional problems^23^ and a high degree of association between aggression and conditions such as anxiety and depression,^24^ the potential moderating role of comorbid internalizing problems in aggressive behavior in children and adolescents with ADHD has been largely ignored. Hence, when exploring the association between ADHD and aggression, it would seem important to consider comorbid problems.

With regard to sex differences in aggression and ADHD, research suggests that similar to boys, girls with ADHD are more likely to exhibit aggression than their peers^5^ and that girls with ADHD and comorbid CD and oppositional defiant disorder tend to exhibit more overt and relational aggression than girls with ADHD only.^25^ At the same time, girls with ADHD are more likely to exhibit inattention symptoms, whereas aggression is more commonly related to the hyperactivity/impulsivity domain.^5^ Furthermore, while the rates of comorbid internalizing problems in girls (compared with boys) with ADHD tend to be higher (especially in adolescence), their physical aggression and other externalizing behaviors may be lower.^26^^,^^27^ At the same time, compared with girls without ADHD, girls with ADHD are more likely to meet the criteria for externalizing relative to internalizing comorbid disorders.^28^ Hence, it has been suggested that there may be sex differences in the association of ADHD with aggressive behavior.^5^ This issue, however, needs to be explored further.

Given the substantial impact that aggressive behavior can have on the psychosocial functioning and future adjustment of children with ADHD, it has been suggested that aggression should be considered as a potential generalized marker of ADHD severity.^29^ Yet, a number of questions regarding the association between ADHD and aggression remain unanswered, including the association of ADHD with different subtypes of aggression, the presence of potential sex differences in these associations, and the extent to which potential associations may be impacted by comorbid conduct and emotional problems. Therefore, the purpose of the present study was to investigate the associations between ADHD symptoms and the cognitive, behavioral, and affective aspects of aggression, while controlling for comorbid conduct and emotional problems. We also aimed to explore whether there are any moderating effects of sex or conduct and emotional problems on the associations between ADHD symptoms and aggression.

## Method

### Procedure

The present study was carried out in the northern Russian city of Arkhangelsk. The city has a population of 356,000, the overwhelming majority of whom are ethnically Russian (98%), while the city’s socioeconomic status (SES) is in the low-to-average range for Russia. To obtain a representative sample, a randomized selection process was used to select schools in the 4 major districts of the city. All the schools approached agreed to participate in the survey. Approximately 10% of the city’s students were recruited in the study. The students and their parents were informed about the study before it began and that study participation was voluntary and that both parents (on behalf of their children) and children had the right to refuse to participate. Assessment was conducted in randomly selected classes within each school. Students who did not want to participate in the survey were provided with alternative activities. Written informed consent was provided by all participants. The study was approved by the institutional review committee at the Northern State Medical University, Arkhangelsk, Russia.

### Participants

A total of 2,892 (96.4% of the total distributed) questionnaires were collected. Participants outside the target age range of 13 to 17 years (n = 45) were excluded. For participants who lacked data for any of the continuous variables (n = 448), multiple imputation was conducted with the expectation-maximization algorithm in IBM SPSS Version 28 (IBM Corp., Armonk, New York) with 25 maximum iterations. Participants who had missing data imputed had lower ratings on hyperactivity/inattention (*t*_2,804_ = 3.12, *p* = .002), had higher ratings on physical (*t*_2,723_ = 5.42, *p* < .001) and verbal (*t*_2,721_ = 3.02, *p* = .003) aggression, were younger (*t*_2,845_ = 2.55, *p* = .011), and were more likely to be male (N = 2,838; χ^2^_1_ = 13.87, *p* < .001). After removing an additional 9 adolescents who did not provide information on sex, the analytic sample comprised 2,838 adolescent students (57.9% female and 42.1% male; mean [SD] age = 14.89 [1.12] years).

### Measures

The school-based survey used in the current study has been used extensively in previous research and includes both scales already available in the literature that have been used with similar populations and new scales developed specifically for this study, which have been subsequently validated.^30^^,^^31^ Descriptive statistics of the scale variables used in the current study are presented in Table S1, available online.

#### ADHD Symptoms

ADHD symptoms were assessed using the hyperactivity/inattention subscale of the Strengths and Difficulties Questionnaire (SDQ), a shorter substitute for the 18-item ADHD symptom list.^32^ This subscale consists of 5 statements (eg, “I am restless, I cannot stay still for long,” “I am easily distracted, I find it difficult to concentrate”), rated on a 3-point scale including the responses “not true” (0), “somewhat true” (1), or “certainly true” (2), with the total score ranging from 0 to 10. The scale has a good ability to discriminate ADHD from other diagnoses.^33^ In this study we used the recommended cutoff of ≥7, suggested in epidemiological research for identifying ADHD.^34^^,^^35^ The hyperactivity/inattention subscale was further validated in a subsample of the study group with the teacher-rated version of the ADHD Rating Scale-IV,^36^ in which participants with the score of ≥7 on the SDQ also had higher teacher ratings of ADHD symptoms (n = 521; mean = 10.93 vs 8.15; *t* = 2.31, *p* = .022, *df* = 519).

#### Conduct Problems and Emotional Problems

Conduct problems and emotional problems were measured using corresponding scales from the SDQ. Each scale consists of 5 statements that screen for conduct problems (eg, “I take things that are not mine from home, school or elsewhere” and “I am often accused of lying or cheating”) and for emotional problems (eg, “I worry a lot” and “I am often unhappy, down-hearted or tearful”) respectively. The scales have shown a good ability to identify the respective psychiatric diagnoses.^32^ As with the hyperactivity/inattention subscale, we aimed to use the recommended cutoffs (≥7 on the emotional problem scale and ≥5 on the conduct problem scale). However, because the sample had lower ratings on emotional problems, we lowered the cutoff to ≥6 points for this scale to include all the adolescents within the abnormal (90th percentile) range. Both subscales were hence dichotomized in an attempt to more clearly demarcate the levels of clinically significant symptoms.

#### Socioeconomic Status

As a proxy for SES, we used student reports on their parents’ education and employment status,^30^ with college education or above scored as 1 (vs 0) and employment status scored as 1 (unemployed = 0), reported separately for each parent. Thus, the possible score could range from 0 to 4, with higher scores indicating higher SES. For single-parent households, the score was divided by 2 to adjust for the lower potential maximum scores.

#### Trait Anger

Trait anger was assessed using the 10-item STAXI Trait Anger subscale,^37^ which assesses an individual’s general tendency to experience anger emotionally, either in the absence of a direct provocation or as a result of specific triggers, such as criticism or unfair treatment by others (eg, “I am a hot-headed person,” “I have a fiery temper,” “I get angry when I’m slowed down by others’ mistakes”). The students rated items using a 4-point scale ranging from almost never (1) to almost always (4). The possible total score ranges from 10 to 40, with higher scores indicating more trait anger. The scale has previously demonstrated excellent internal consistency in Russian populations.^30^^,^^38^ In the present sample, Cronbach α for the scale was .89.

#### Anger Rumination

Anger rumination was evaluated with the Anger Rumination Scale (ARS),^9^ which assesses a person’s tendency to focus or ruminate on thoughts and memories of anger experiences, thereby leading the person to experience the continuation of anger after the episode is over. The ARS consists of 17 items (eg, “After an argument is over, I keep fighting with this person in my imagination,” “When someone makes me angry, I can’t stop thinking about how to get back at them”). Items are rated on a 4-point scale, from “almost never” (1) to “almost always” (4), with the total score range of 17 to 68 and higher scores indicating more anger rumination. The ARS has demonstrated adequate internal consistency and test-retest reliability as well as convergent and discriminant validity.^9^ In the present sample, Cronbach α was .92 for the scale.

#### Aggressive Beliefs

Aggressive beliefs^31^ were measured using a 6-item measure that assesses beliefs that legitimize physical aggression as a behavioral style in response to interpersonal provocation or conflict (eg, “Bullying others makes you feel confident and strong,” “Sometimes when you are angry, to strike somebody is a completely normal reaction”). Items were rated on a 4-point scale from “completely disagree” (1) to “completely agree” (4), with a possible score range of 6 to 24. Cronbach α for this measure was .66.

#### Proactive, Physical, and Verbal Aggression

Proactive, physical, and verbal aggression were assessed using a 15-item scale that inquires about the frequency of students’ aggressive behavior during the past 30 days.^30^^,^^31^ The scale includes 5 items related to proactive aggression (eg, “Bullied somebody just for the fun of it,” “Deliberately made others cry”), 5 items related to physical aggression (eg, “Slapped someone across the head,” “Punched someone in a fight”), and 5 items related to verbal aggression (eg, “Threatened or intimidated others,” “Called others names”). The items were rated using a 4-point scale that ranged from “almost never” (0) to “five or more times” (3). The total score for each form of aggression ranged from 0 to 15 with higher scores indicating more aggression. In this study, Cronbach α was .78 for proactive aggression, .79 for physical aggression, and .78 for verbal aggression.

#### Social Aggression

Social aggression^31^ was evaluated with 9 items (eg, “spread rumors/gossip,” “set up situations in which the person looked stupid”) using a 4-point scale that ranged from “almost never” (1) to “almost always” (4), with a possible range of 9 to 36 and higher scores indicating increased social aggression. The scale has been previously used with Russian populations, demonstrating good psychometric properties, and it represents a separate factor, distinctly different from physical and verbal aggression.^31^ Cronbach α for this measure was .81.

### Statistical Analyses

Data were analyzed using IBM SPSS Version 28. Possible sex differences in anger and aggression ratings were assessed using a t test for independent samples and χ^2^ test. The normality of the distributions of the variables was explored. All scales showed a right-skewed gamma distribution and hence were not normally distributed. The Akaike information criterion indicated that a gamma distribution with log link had the best model fit. Therefore, generalized linear models with gamma as the distribution and log as the link function were used to assess differences in various aspects of aggression in male and female participants, with or without clinically significant levels of ADHD symptoms (a score of ≥7 on the hyperactivity/inattention subscale of the SDQ). The analysis was adjusted for sex, age, and SES in the first model and additionally adjusted for clinically significant levels of conduct problems and emotional problems in the second model. As a post hoc analysis, we recalculated the fully adjusted generalized linear model to assess the moderating/interaction effects of sex and comorbid symptoms by ADHD in separate models. Thus, we used 2 × 2 designs for the aggression scales: 2 (clinical vs subclinical ADHD symptoms) × 2 (sex), 2 (clinical vs subclinical ADHD symptoms) × 2 (clinical vs subclinical emotional symptoms), or 2 (clinical vs subclinical ADHD symptoms) × 2 (clinical vs subclinical conduct problems). In the analyses, two-tailed tests with a *p* value < .05 were considered statistically significant. Results are presented as means and standard deviations and, for individual outcomes, B values.

## Results

### Comparisons by Group and Sex

Descriptive statistics of the study variables are presented in Table 1 and Table S1, available online. Correlations between the study variables are presented in Table S2, available online. A total of 299 adolescents (10.5% of the sample, including 192 females [11.7%] and 107 males [9%]) scored ≥7 on the hyperactivity/inattention subscale of the SDQ, indicating the presence of clinically significant levels of ADHD symptoms. For conduct problems, 445 adolescents scored ≥5 (15.7% of the sample, including 221 females [13.4%] and 224 males [18.8%]), and for emotional problems, 392 adolescents scored ≥6 (13.8% of the sample, including 291 females [17.7%] and 101 males [8.5%]). Females scored higher than males on the measures of anger rumination (*t* = 4.60, *p* < .001, *df* = 2836) and trait anger (*t* = 6.55, *p* < .001, *df* = 2836), with emotional problems also more prevalent in females (χ^2^ = 49.62, *p* < .001, *df* = 1), whereas males scored higher on proactive aggression (*t* = 8.46; *p* < .001, *df* = 2836), aggressive beliefs (*t* = 9.95, *p* < .001, *df* = 2836), and physical (*t* = 18.02, *p* < .001, *df* = 2836) and verbal (*t* = 8.12, *p* < .001, *df* = 2836) aggression, with the prevalence of conduct problems also higher in males compared with females (χ^2^ = 14.79, *p* < .001, *df* = 1).

**Table 1:** Study Variables by Sex and Attention-Deficit/Hyperactivity Disorder (ADHD) Symptoms

	All participants (N = 2,838)		Clinically significant levels of ADHD (≥7 points) (n = 299)		No ADHD (<7 points) (n = 2,539)	
	Mean	(SD)	Mean	(SD)	Mean	(SD)
Trait anger						
F	21.71	(6.83)	27.22	(6.68)	20.99	(6.51)
M	20.03	(6.69)	24.66	(7.48)	19.57	(6.43)
Anger rumination						
F	32.22	(9.79)	35.37	(11.31)	31.81	(9.49)
M	30.44	(10.51)	35.84	(13.08)	29.90	(10.08)
Aggression beliefs						
F	14.68	(3.23)	15.51	(3.34)	14.58	(3.20)
M	15.96	(3.46)	16.98	(3.69)	15.86	(3.42)
Proactive aggression						
F	3.86	(3.22)	5.94	(3.73)	3.58	(3.04)
M	5.01	(3.80)	6.86	(4.10)	4.82	(3.72)
Physical aggression						
F	2.09	(2.65)	3.66	(3.72)	1.88	(2.41)
M	4.44	(3.89)	5.82	(4.04)	4.30	(3.86)
Verbal aggression						
F	3.88	(3.11)	6.00	(3.65)	3.60	(2.92)
M	5.01	(4.04)	7.17	(4.15)	4.80	(3.97)
Social aggression						
F	13.18	(3.92)	14.56	(4.24)	12.99	(3.84)
M	13.26	(4.69)	16.77	(6.76)	12.91	(4.28)
	**n**	**(%)**	**n**	**(%)**	**n**	**(%)**
Emotional problems^a^						
F	291	(17.7)	43	(22.4)	248	(17.1)
M	101	(8.5)	21	(19.6)	80	(7.4)
Conduct problems^a^						
F	221	(13.4)	72	(37.5)	149	(10.3)
M	224	(18.8)	46	(43.0)	178	(16.4)

Note: The values presented are not adjusted for the other covariates. F = females; M = males.

a Emotional problems ≥6 points on the Strengths and Difficulties Questionnaire (SDQ) subscale; conduct problems ≥5 points on the SDQ subscale

### Association Between ADHD Symptoms and Aggression, Not Adjusting for Comorbidity

The estimates of association between ADHD symptoms and aggression are presented in Table 2. Clinically significant levels of ADHD symptoms predicted all components of aggression. There was also an effect for sex, with higher levels of trait anger and anger rumination among females and higher levels of aggressive beliefs, proactive aggression, and physical and verbal aggression among males. Furthermore, increasing age was associated with more trait anger, anger rumination, and social aggression, but fewer aggressive beliefs and physical aggression. When including an interaction effect for ADHD × sex, we found a main effect where males with ADHD symptoms displayed a larger increase than females in social aggression.

**Table 2:** Associations Between Attention-Deficit/Hyperactivity Disorder (ADHD) Symptoms and Different Components of Aggression

	Trait anger		Anger rumination		Aggressive beliefs		Proactive aggression		Physical aggression		Verbal aggression		Social aggression	
	*B*	(95% CI)	*B*	(95% CI)	*B*	(95% CI)	*B*	(95% CI)	*B*	(95% CI)	*B*	(95% CI)	*B*	(95% CI)
ADHD	0.25	(0.21, 0.29)∗∗∗	0.13	(0.09, 0.17)∗∗∗	0.07	(0.04, 0.09)∗∗∗	0.45	(0.30, 0.61)∗∗∗	5.25	(2.15, 8.35)∗∗∗	0.47	(0.32, 0.63)∗∗∗	0.17	(0.13, 0.21)∗∗∗
Sex, male	−0.07	(−0.09, −0.05)∗∗∗	−0.05	(−0.08, −0.03)∗∗∗	0.08	(0.07, 0.10)∗∗∗	0.29	(0.19, 0.38)∗∗∗	0.58	(0.38, 0.79)∗∗∗	0.28	(0.18, 0.38)∗∗∗	0.01	(−0.01, 0.03)
Age	0.01	(0.00, 0.03)∗∗	0.02	(0.01, 0.03)∗∗	−0.01	(−0.02, −0.00)∗	0.01	(−0.03, 0.05)	−0.28	(−0.37, −0.19)∗∗∗	0.01	(−0.04, 0.05)	0.01	(0.00, 0.02)∗
SES	−0.01	(−0.02, 0.00)	−0.01	(−0.02, 0.00)	−0.00	(−0.01, 0.00)	0.02	(−0.02, 0.06)	0.24	(0.14, 0.33)∗∗∗	0.02	(−0.03, 0.06)	−0.01	(−0.02, 0.00)
ADHD × sex^a^	−0.03	(−0.10, 0.05)	0.08	(−0.00, 0.16)	0.00	(−0.05, 0.06)	−0.15	(−0.47, 0.17)	−0.34	(−0.78, 0.10)	−0.11	(−0.43, 0.21)	0.15	(0.08, 0.22)∗∗∗

Note: B = unstandardized regression coefficient; SES = socioeconomic status.

∗*p* < .05; ∗∗*p* < .01; ∗∗∗*p* < .001.

a The interaction effect was assessed in a separate model, also adjusting for the other variables in the model.

### Association Between ADHD Symptoms and Aggression, Additionally Adjusting for Comorbidity

**Figure 1:**
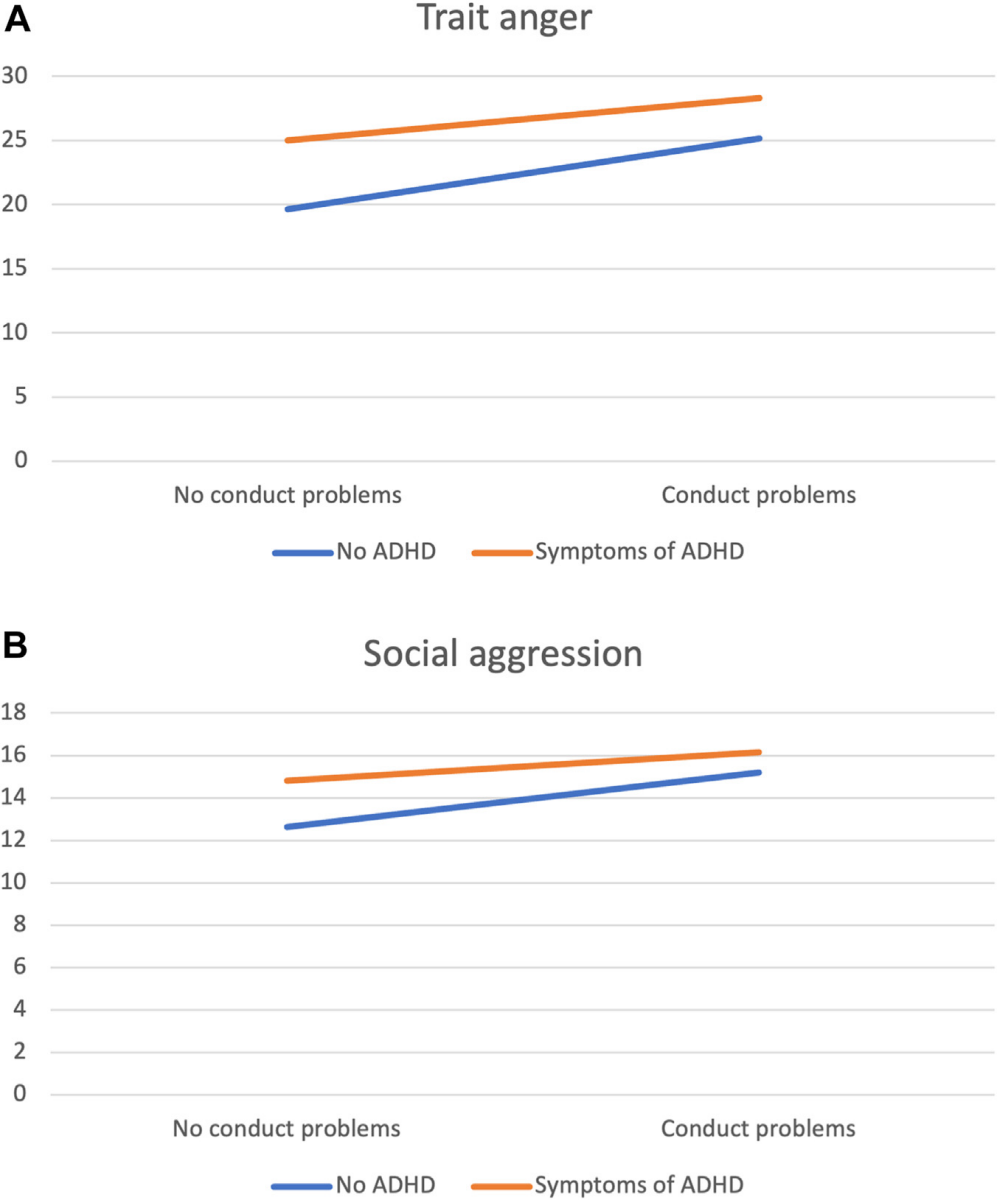
Trait Anger and Social Aggression as a Function of Clinically Significant Levels of Attention-Deficit/Hyperactivity Disorder (ADHD) and Conduct Problems ***Note:** Trait anger and social aggression scores are on the y-axis. (A) Trait anger as a function of clinically significant levels of ADHD and conduct problems. (B) Social aggression as a function of clinically significant levels of ADHD and conduct problems*.

After adjusting for clinically significant levels of emotional problems and conduct problems, the effect for clinically significant levels of ADHD symptoms remained for all components of aggression (Table 3). Clinically significant levels of emotional problems were associated with higher levels of trait anger, anger rumination, and social aggression, but decreasing levels of proactive aggression. Clinically significant levels of conduct problems were associated with increasing levels of all forms of aggression. The effects for sex and age remained significant. The interaction effect of ADHD symptoms × sex on social aggression remained. Furthermore, there were interaction effects of ADHD symptoms and conduct problems on trait anger and social aggression, where participants with ADHD symptoms had a lower increase in trait anger and social aggression when also having conduct problems compared with participants without clinical levels of ADHD (Figure 1).

**Table 3:** Associations Between Attention-Deficit/Hyperactivity Disorder (ADHD) Symptoms and Different Components of Aggression While Adjusting for Comorbid Symptoms

	Trait anger		Anger rumination		Aggressive beliefs		Proactive aggression		Physical aggression		Verbal aggression		Social aggression	
	*B*	(95% CI)	*B*	(95% CI)	*B*	(95% CI)	*B*	(95% CI)	*B*	(95% CI)	*B*	(95% CI)	*B*	(95% CI)
ADHD	0.18	(0.15, 0.22)∗∗∗	0.07	(0.04, 0.11)∗∗∗	0.04	(0.02, 0.07)∗∗	0.35	(0.20, 0.51)∗∗∗	0.40	(0.18, 0.61)∗∗∗	0.37	(0.21, 0.52)∗∗∗	0.12	(0.09, 0.16)∗∗∗
Sex, male	−0.07	(−0.10, −0.05)∗∗∗	−0.04	(−0.07, −0.02)∗∗∗	0.08	(0.06, 0.10)∗∗∗	0.24	(0.15, 0.34)∗∗∗	0.76	(0.62, 0.89)∗∗∗	0.25	(0.15, 0.34)∗∗∗	0.01	(−0.02, 0.03)
Age	0.01	(0.00, 0.02)∗∗	0.02	(0.01, 0.03)∗∗	−0.01	(−0.02, −0.00)∗	0.01	(−0.03, 0.06)	−0.07	(−0.13, −0.01)∗	0.01	(−0.03, 0.05)	0.01	(0.00, 0.02)∗
SES	−0.01	(−0.02, 0.00)	−0.01	(−0.02, 0.00)	−0.00	(−0.01, 0.00)	0.02	(−0.02, 0.07)	−0.01	(−0.07, 0.05)	0.02	(−0.03, 0.06)	−0.01	(−0.02, 0.00)
Emotional problems	0.11	(0.08, 0.14)∗∗∗	0.20	(0.17, 0.24)∗∗∗	0.00	(−0.03, 0.02)	−0.15	(−0.29, −0.01)∗	−0.06	(−0.25, 0.13)	−0.03	(−0.17, 0.11)	0.08	(0.04, 0.11)∗∗∗
Conduct problems	0.22	(0.19, 0.25)∗∗∗	0.14	(0.11, 0.17)∗∗∗	0.08	(0.06, 0.10)∗∗∗	0.48	(0.34, 0.61)∗∗∗	0.65	(0.47, 0.83)∗∗∗	0.47	(0.34, 0.61)∗∗∗	0.16	(0.13, 0.19)∗∗∗
ADHD × sex^a^	−0.03	(−0.11, 0.04)	0.06	(−0.01, 0.14)	0.01	(−0.05, 0.06)	−0.15	(−0.46, 0.17)	−0.34	(−0.77, 0.10)	−0.10	(−0.42, 0.22)	0.15	(0.07, 0.22)∗∗∗
ADHD × emotional problems^a^	0.01	(−0.08, 0.10)	0.05	(−0.04, 0.14)	0.04	(−0.02, 0.11)	0.14	(−0.24, 0.52)	0.18	(−0.35, 0.71)	−0.01	(−0.40, 0.37)	0.06	(−0.02, 0.15)
ADHD × conduct problems^a^	−0.11	(−0.19, −0.03)∗∗	0.01	(−0.08, 0.09)	−0.04	(−0.10, 0.02)	−0.29	(−0.62, 0.05)	−0.49	(−8.21, 7.22)	−0.30	(−0.64, 0.03)	−0.09	(−0.16, −0.01)∗

Note: B = unstandardized regression coefficient; SES = socioeconomic status.

∗*p* < .05; ∗∗*p* < .01; ∗∗∗*p* < .001.

a The interaction effect was assessed in a separate model, also adjusting for the other variables in the model.

## Discussion

The purpose of this cross-sectional study was to assess whether ADHD symptoms are associated with different components of aggression in adolescents, while also exploring any moderating effects of sex as well as comorbid conduct and emotional problems. Results showed that self-rated clinically significant levels of ADHD symptoms were associated with the cognitive, behavioral, and affective components of aggression and that these associations remained significant after adjusting for emotional and conduct problems. Females had higher levels of trait anger and anger rumination, whereas males had higher levels of aggressive beliefs, proactive aggression, and physical and verbal aggression. An interaction effect was observed for ADHD × sex, where males (compared with females) with ADHD symptoms reported higher levels of social aggression. Finally, an interaction effect was found for ADHD × conduct problems, where participants with clinically significant levels of ADHD symptoms (compared with those without) had a less significant increase in trait anger and social aggression when including conduct problems in the analytic model, hence suggesting that individuals with ADHD symptoms, who already had substantially higher levels of aggression, were less impacted by the presence of comorbid conduct problems.

Our findings on the association between ADHD symptoms and aggression support previous reports of aggression as being an important associated feature of ADHD.^5^ Clinical levels of ADHD symptoms were associated with all components of aggression. As reduced impulse control is a core diagnostic characteristic of ADHD, while the disorder has also been linked to difficulties in regulating emotions,^39^^,^^40^ which may further exacerbate behavioral and/or emotional reactions that are not adaptive or goal oriented,^41^ ADHD has been mostly associated with reactive aggression.^4^^,^^5^ Although we did not explicitly examine reactive aggression in this study, trait anger reflects an individual’s disposition to become angry and to have an angry temperament, thus corroborating previous research on this association. Interestingly, there was also an effect of ADHD symptoms on proactive aggression, a more goal-oriented, cold-blooded facet of aggression that is not generally associated with ADHD^5^^,^^12^^,^^13^ and that in adolescents with ADHD has been hypothetically explained by the influence of antisocial peers.^12^ Similar to Murray *et al.*,^42^ this finding suggests that adolescents with ADHD symptoms may also express their aggressive tendencies in a more deliberate behavioral fashion.

Further, ADHD symptoms were also associated with such cognitive components of aggression as anger rumination and aggressive beliefs. Although studies investigating anger rumination in relation to ADHD are scarce, a tendency to ruminate about a provocation has been linked to an increased likelihood of aggressive behavior occurring as a result of a minor annoyance.^43^ It has been further suggested that anger rumination may be responsible for the persistence of aggressive behavior over time in relation to higher ADHD symptoms.^11^ Rumination has also been regarded as a maladaptive emotion regulation strategy, and, as such, has been associated with emotional distress, anxiety, and depression,^44^ all of which have also been linked to aggression.^24^

The association between clinically significant levels of ADHD symptoms and all the components of aggression remained significant even after adjusting for comorbid symptoms. Given the serious problems with social adjustment observed in individuals with ADHD, these findings support the notion that aggression should be considered an important marker of ADHD severity. Notably, these associations remained even after adjusting for conduct problems, which was the comorbidity most strongly associated with trait anger; aggressive beliefs; and proactive, physical, verbal, and social aggression. Indeed, in line with our findings, research suggests that even after controlling for comorbid CD and oppositional defiant disorder, children with ADHD remain significantly more aggressive than controls.^19^^,^^45^ Interestingly, the association remained significant even for proactive aggression, which has been primarily linked to CD rather than ADHD.^5^ Further, the presence of comorbid conduct problems did not significantly increase aggression scores among those with ADHD symptoms. This finding contradicts previous reports that the combination of ADHD symptoms and conduct problems results in an additive effect and as such could be regarded as a taxonomically distinct group.^46^ These differences may be explained by research findings suggesting that while there is a substantial overlap between the conduct problems and ADHD domains, they also differ in several important respects, including in both their underlying factors (a greater role of social factors in CD compared with ADHD) and functional impairment (more cognitive and achievement deficits in individuals with ADHD).^21^

Although there is a paucity of research on the role of comorbid internalizing problems in aggressive behavior in adolescents with ADHD, our findings indicate that while such symptoms had significant associations with different aspects of aggression, they did not moderate the association between ADHD symptoms and aggression, hence indicating a more direct contribution of ADHD to aggression. The observed sex differences in aggression, with females reporting higher levels of trait anger and anger rumination and males reporting higher levels of aggressive beliefs, proactive aggression, and physical and verbal aggression, are in line with previous research. Studies indicate that male adolescents are more likely to report physical aggression^47^ and to exhibit proactive aggression^48^ than females, whereas no sex differences have been found in terms of social aggression,^47^ and self-rated anger has been more common among female adolescents compared with males.^49^ In addition, an interaction effect for clinically significant levels of ADHD symptoms × sex suggests that males (compared with females) with ADHD symptoms report higher levels of social aggression. These findings suggest that a high level of ADHD symptoms may further exacerbate the existing sex-specific differences in certain components of aggression.

This study has several strengths, including using data from a large sample of community-based adolescents and being able to examine ADHD symptoms in relation to different forms of aggression. Nonetheless, there are several limitations that should also be mentioned. We relied on self-reported ratings that can result in bias, such as recall bias and social desirability bias. Although teacher reports on ADHD symptoms used in this study supported the use of self-reports for the identification of clinically significant ADHD symptoms, using data from other information sources, such as parents or structured diagnostic interviews and medical records, would have improved confidence in our findings. It should be noted that although the SDQ is a widely used and established measure of mental health problems, the psychometric properties of the subscales have often not been optimal,^32^ including those in the Russian version,^50^ likely due to the use of a few broad items and reverse scoring. In particular, the conduct problems subscale items were poorly interrelated, although the ordinal α value was increased when dropping the reversed coded item. However, we used dichotomized scores on the SDQ, where we defined the top 10% of individuals as having clinically significant or abnormal levels of ADHD symptoms, as recommended by Goodman^32^ and in an attempt to more clearly demarcate the levels of clinically significant symptoms to identify adolescents who would benefit from further assessment or intervention. Although this analytic strategy may have limited statistical power and does not take into account the distribution of these symptoms in the sample, using dichotomized variables facilitated the interpretation of the interaction analyses, and given the large sample size, we deemed that there was sufficient power to conduct the analyses. The aggression scales had good psychometric properties, with the exception of the aggressive beliefs scale, where the internal reliability was lower, indicating poorer interrelatedness between the items.

We also lacked data on whether any of the students had received psychiatric diagnoses and whether any of them were being treated for psychiatric symptoms, including using ADHD medication, which might have been important for the observed associations. As our study used cross-sectional data, it was not possible to determine the temporal nature of the associations, or establish causality. In future research, longitudinal designs should be applied to better understand trajectories of aggressive behaviors in adolescents with ADHD, while adjusting for comorbid symptoms and any sex-specific effects. Finally, as the study was limited to one geographical region of Russia, further research is needed to establish the generalizability of our findings.

In conclusion, ADHD seems to have an impact on all components of aggression, and there is a sex-specific difference in the association with social aggression. The results of this study further support the notion that ADHD symptoms are relevant for aggression independent of comorbidity. These findings are alarming given that aggression may further negatively impact psychosocial functioning in children with ADHD,^3^^,^^4^ hence highlighting the importance of considering aggression when evaluating the severity of ADHD as well as treatment methods such as the type of medication. Our findings also suggest the importance of taking into account comorbid emotional and conduct problems, which by themselves may increase the risk for aggression. In future studies, ADHD symptoms should be assessed more comprehensively, with assessment including the use of diagnostic interviews, when exploring these associations.
