# Supplementary material for: Attention-Deficit/Hyperactivity Disorder Symptoms and Anger and Aggression in Russian Adolescents
Source: JAACAP Open. 2024 Feb 16;3(1):126–36. doi: 10.1016/j.jaacop.2024.01.006 (PMC11914921; doi:10.1016/j.jaacop.2024.01.006)
Supplement: Table S1 [file mmc1.docx]

**Supplementary Table 1 Descriptive statistics of the scale variables used in the study**

|  | Mean score (SD) | min, max score | Coefficient alpha |
| --- | --- | --- | --- |
| Trait anger | 21.00 (6.82) | 10, 40 | .89^a^ |
| Anger rumination | 31.47 (10.14) | 17, 68 | .92^a^ |
| Aggressive beliefs | 15.22 (3.39) | 6, 24 | .66^a^ |
| Proactive aggression | 4.34 (3.52) | 0, 15 | .78^a^ |
| Physical aggression | 3.08 (3.44) | 0, 15 | .79^a^ |
| Verbal aggression | 4.36 (3.58) | 0, 15 | .78^a^ |
| Social aggression | 13.21 (4.26) | 9, 36 | .81^a^ |
| ADHD symptoms  ≥7 n, % | 3.90 (2.03)  299, 10.7% | 0, 10 | .62^b^ |
| Emotional problems  ≥6 n, % | 2.81 (2.26)  392, 13.8% | 0, 10 | .79^b^ |
| Conduct problems  ≥5 n, % | 2.92 (1.59)  445, 15.7% | 0, 10 | .49^b^ |

***Note:*** ADHD = Attention-deficit/hyperactivity disorder; SD = Standard deviation

^a^ Calculated using Cronbach alpha; ^b^ calculated with polychoric ordinal alpha
