# Supplementary material for: Attention-Deficit/Hyperactivity Disorder Symptoms and Anger and Aggression in Russian Adolescents
Source: JAACAP Open. 2024 Feb 16;3(1):126–36. doi: 10.1016/j.jaacop.2024.01.006 (PMC11914921; doi:10.1016/j.jaacop.2024.01.006)
Supplement: Table S2 [file mmc2.docx]

**Supplementary Table 2 Correlations between the study variables using Phi-coefficient for dichotomous variables, Point-Biserial Correlation between continuous and dichotomous variables, and Spearman’s rho between continuous variables**

|  | ADHD | Em probl | Cond probl | Sex (male) | Age | SES | Trait agg | Anger Rum | Agg beliefs | Proact agg | Phys agg | Verbal agg |
| --- | --- | --- | --- | --- | --- | --- | --- | --- | --- | --- | --- | --- |
| ADHD |  |  |  |  |  |  |  |  |  |  |  |  |
| Em probl | .08*** |  |  |  |  |  |  |  |  |  |  |  |
| Cond probl | .22*** | .12*** |  |  |  |  |  |  |  |  |  |  |
| Sex (male) | -.04* | -.13*** | .07*** |  |  |  |  |  |  |  |  |  |
| age | .02 | .05** | -.01 | -.08*** |  |  |  |  |  |  |  |  |
| SES | -.01 | -.01 | -.01 | -.03 | .05** |  |  |  |  |  |  |  |
| Trait agg | .27*** | .19*** | .32*** | -.12*** | .06*** | -.03 |  |  |  |  |  |  |
| Anger rum | .14*** | .28*** | .21*** | -.09*** | .08*** | -.04 | .44*** |  |  |  |  |  |
| Agg beliefs | .08*** | -.00 | .16*** | .19*** | -.07*** | -.03 | .21*** | .09*** |  |  |  |  |
| Proact agg | .19*** | -.04* | .29*** | .16*** | .02 | .04* | .33*** | .15*** | .29*** |  |  |  |
| Phys agg | .14*** | -.03 | .30*** | .34*** | -.10*** | -.01 | .25*** | .12*** | .35*** | .60*** |  |  |
| Verbal agg | .20*** | .00 | .29*** | .16*** | .01 | .03 | .40*** | .23*** | .30*** | .73*** | .67*** |  |
| Social agg | .17*** | .12*** | .23*** | .01 | .05* | -.01 | .38*** | .33*** | .17*** | .34*** | .26*** | .36*** |

***Note:*** ADHD = Attention-deficit/hyperactivity disorder; Agg beliefs = Aggressive beliefs; Anger rum = Anger rumination; Cond probl = Conduct problems; Em probl = Emotional problems; Phys agg = Physical aggression; Proact agg = Proactive aggression; SES = Socioeconomic status; Trait agg = Trait aggression; Verbal agg = Verbal aggression;

*p<.05. **p<.01. ***p<.001.
